# An Integrated Method for CFRP Drilling Quality Prediction and Multi-Objective Optimization

**DOI:** 10.3390/ma19102101

**Published:** 2026-05-16

**Authors:** An Ping, Yibin Zha, Yichen Li, Yiwen Jiang, Kailong He, Xuehui Gan, Hui Zhang, Jin Liu

**Affiliations:** 1 College of Mechanical Engineering, Donghua University, Shanghai 201620, China; 1229070@mail.dhu.edu.cn (A.P.); 1219077@mail.dhu.edu.cn (Y.Z.); 1249074@mail.dhu.edu.cn (Y.J.); 2231081@mail.dhu.edu.cn (K.H.); 2 College of Fashion and Design, Donghua University, Shanghai 201620, China; 18639244965@163.com; 3 College of Material Science and Engineering, Donghua University, Shanghai 201620, China; zhanhui@dhu.edu.cn (H.Z.); lj@dhu.edu.cn (J.L.)

**Keywords:** CFRP drilling, processing quality, multi-objective optimization

## Abstract

Carbon fiber-reinforced polymer (CFRP) is extensively applied in aerospace and rail transportation industries, and the quality of CFRP joint holes is crucial for ensuring joint performance and service reliability. However, CFRP drilling involves complex interactions among drilling parameters, tool geometry, and multiple quality indicators, making it difficult to accurately predict drilling quality and identify optimal process parameters. In this study, drilling experiments using twist drills and dagger drills were conducted to analyze the relationships among drilling parameters, drilling physical indicators, and drilling quality indicators. Compared to the twist drill, the dagger drill maintained lower thrust force and cutting temperature, and reduced the delamination factor, burr factor, and drilling wall roughness by 20.6%, 95.5%, and 81.1% on average, respectively. To consider the effect of different drilling indicators on the quality of CFRP drilling holes, a comprehensive fuzzy evaluation prediction model FCE-NN for drilling quality was proposed. The average prediction accuracy reached 91.3% and the drilling indicators FCE were output. The NSGA-II algorithm was employed, and an entropy-weighted TOPSIS method integrated with fuzzy comprehensive evaluation (FCE) was used to rank the Pareto-optimal solutions, thereby achieving multi-objective optimization of the thrust factor, delamination factor, and machining efficiency.

## 1. Introduction

Carbon fiber-reinforced polymer (CFRP), owing to its outstanding material performance, has emerged as a vital material for achieving structural lightweighting and performance improvement in aerospace and rail vehicles [1,2,3]. When CFRP components are assembled, mechanical fastening through drilled holes, such as riveting and bolted joints, remains the predominant joining method [4,5]. However, CFRP is susceptible to drilling defects, in particular entry and exit delamination, burrs, and high hole-wall surface roughness [6,7,8]. These defects may lead to joint-hole failure or structural damage, thereby seriously threatening the safety and service reliability of aerospace and rail vehicles [9]. Therefore, suppressing drilling defects, improving hole quality, and optimizing high-quality drilling parameters have attracted significant attention in CFRP applications.

Initially, the optimization of drilling quality mainly focused on investigating drilling processes through experimental approaches. Jia et al. studied the drilling quality under different drilling parameters. Their study showed that drilling quality is the result of the combined effects of thrust force and temperature [10]. Urresti et al. conducted drilling experiments on aerospace CFRP components using tools with different geometries. They investigated the damage quality during tool entry and exit, identified the optimal cutting geometry, and analyzed its influence on delamination damage. In addition, drilling quality was monitored using the mean thrust force, the difference between entry and exit force gradients, and the exit energy as evaluation indicators [11,12]. Alcón et al. experimentally investigated CFRP drilling quality under different machining parameters. Their study analyzed the influence of process parameters on hole-wall surface roughness, cylindricity, and hole diameter, and identified the optimal combination of drilling parameters [13]. Zhang et al. performed experiments using twist drills and step drills, and comparatively investigated the occurrence of delamination and burrs under different drilling parameters [14]. Qiu et al. researched the variation of different types of delamination with feed rate in CFRP at a constant spindle speed [15]. Yuan et al. investigated the impact of different process parameters on the surface roughness of composite hole walls [16]. Kim et al. used tools with different geometries to drill CFRP and investigated the influence of tool design and workpiece material on drilling quality [17]. These studies have mainly investigated the optimization and prediction of machining parameters for a single objective [18,19,20], making it difficult to comprehensively reflect the multi-objective coupling effects and the trade-off with machining efficiency in complex drilling processes.

Therefore, many recent studies have shifted their focus toward the multi-objective optimization and prediction of process parameters. Li et al. studied trimming of CFRP using multiple neural network architectures combined with the TOPSIS method. Neural networks were used to establish and predict the relationships between processing parameters and tool wear level, hole wall surface roughness, and tool temperature. TOPSIS was used for multi-objective optimization of cutting conditions to achieve optimal machining performance [21]. Meahdi et al. conducted analyses of the drilling quality of woven CFRP using drill bits with different geometries. A regression-based response surface method and an artificial neural network (ANN) composed of neurons at different hierarchical levels were employed for comparative modeling of drilling quality. Furthermore, a genetic algorithm was utilized to optimize the drilling parameters [22]. Jia et al. studied drilling experiments on CFRP with two different matrix resins. ANOVA method was applied to analyze the effects of drilling parameters on thrust force, maximum cutting temperature, and degree of delamination. The NSGA-II algorithm, in combination with the TOPSIS method, was employed to the Pareto-optimal results and identify the best drilling conditions [23]. Wu et al. studied CF/PEEK under ultrasonic-assisted drilling conditions. ANOVA-based polynomial regression was applied to analyze the relationships between thrust force, material removal rate, and delamination factor. The NSGA-II algorithm was then used to optimize processing parameters with thrust force, material removal rate, and delamination factor as objectives, thereby obtaining the Pareto-optimal solutions [24]. Wang et al. considered spindle speed, feed rate, and feed angle as the main machining parameters, and established an artificial neural network to predict the relationships between drilling parameters, thrust force, and delamination factor. Fuzzy c-means clustering was applied to narrow the range of Pareto front solutions obtained by NSGA-II for more representative results [25].

Although these studies have achieved promising results in optimization and prediction, some limitations still remain. CFRP drilling is inherently a complex machining process involving the coupled effects of tool geometry, drilling parameters, drilling quality indicators, and machining efficiency. Due to the nonlinear relationships and multi-objective conflicts among these factors, optimization based solely on a single defect indicator or on the data structure of multi-objective solutions is insufficient to represent the optimization of drilling parameters from the perspective of overall drilling quality, and it also makes it difficult to achieve the coordinated optimization of comprehensive drilling quality and machining efficiency. In most existing studies, indicators such as *F_D_*, *F_B_*, and *Ra* are treated in parallel, implicitly assuming equal importance among them, which is inconsistent with practical engineering conditions. In addition, existing multi-objective optimization methods based on NSGA-II combined with TOPSIS can obtain the Pareto front. However, the obtained Pareto front still contains many candidate solutions. Most existing studies either manually select representative solutions or determine the ideal solution solely according to the data structure characteristics, often without considering the different influence levels of various drilling-quality indicators. Therefore, how to conduct an engineering-oriented selection from the Pareto-front solutions remains an insufficiently addressed but important challenge in CFRP drilling optimization. To address these limitations, drilling experiments using twist drills and dagger drills were conducted in this study to comparatively analyze the differences in drilling characteristics, and regression models were established to characterize the relationships between drilling parameters and drilling indicators. A comprehensive indicator evaluation model based on FCE was established. FCE can assign different levels of importance to different indicators by incorporating practical engineering judgment and weighting, making the evaluation results more consistent with the actual requirements of CFRP drilling quality control. Based on the fuzzy-rule-based comprehensive evaluation indicator, an FCE-BP neural network prediction model was constructed to predict drilling quality defects and obtain a comprehensive evaluation indicator capable of describing the degree of quality defects. The FCE indicator was further introduced into the re-evaluation process of Pareto solutions, so that the finally selected parameters are not only close to the ideal solution in a mathematical sense, but also more consistent with practical engineering requirements in terms of drilling quality. In addition, a response surface model was developed, in which thrust force, delamination factor, and machining efficiency were taken as optimization objectives, while cutting temperature, burrs, and hole-wall surface roughness were treated as constraints. Multi-objective optimization was then carried out using NSGA-II and entropy-weighted TOPSIS. Finally, the Pareto-front solutions were further re-evaluated and optimized using the FCE index, thereby obtaining the optimal drilling parameters for each tool, which were subsequently validated through experiments.

## 2. Materials and Experiment

### 2.1. Experimental Design

**Figure 1:**
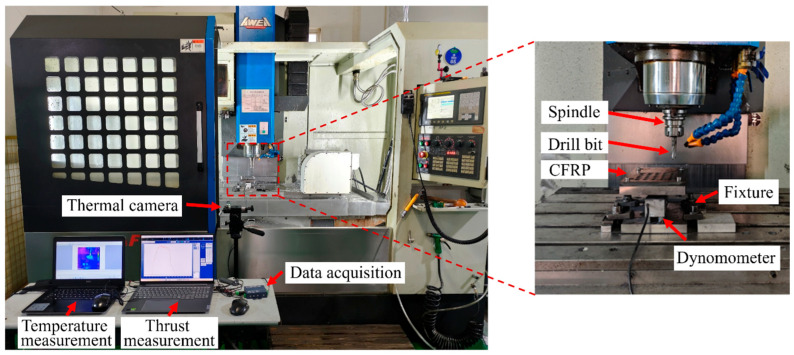
Drilling experiment.

**Figure 2:**
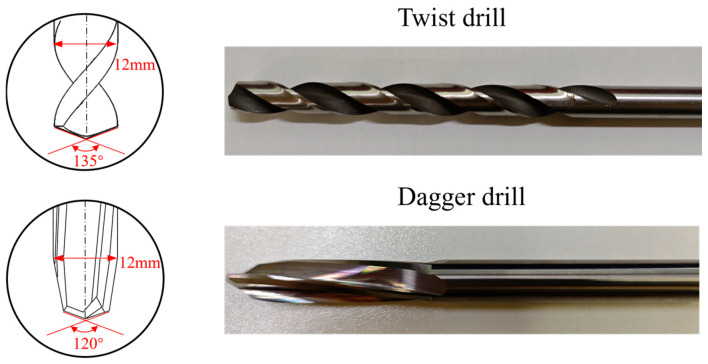
Drill bit type.

The CFPR material plates used in the experiments were composed of T800-12K carbon fibers and an epoxy resin matrix 977, and was provided by CRRC Qingdao Sifang Co., Ltd., Qingdao, China. The CFPR plates had a thickness of 10 mm and were stacked using a lay-up method. Drilling experiments were conducted using twist drills and dagger drills under different drilling parameters to systematically obtain data on drilling physical quantities, quality indicators, and machining efficiency, and to analyze the impact of different parameters on drilling characteristics under different tool geometries. This process not only reveals the differences between the two drill types in terms of damage suppression and efficiency improvement, but also provides the data basis for establishing regression and predictive models between machining parameters and drilling responses, thereby offering theoretical and data support for the construction of objective and constraint functions in subsequent multi-objective optimization, and for the validation of optimization results. The experiments were used on a CNC machine (AF1000), (Taiwan, China) under dry drilling conditions as shown in Figure 1. During the drilling process, the thrust was measured using a dynamometer, and the cutting temperature was measured with a thermal camera. The types of cutting tools used were as follows: twist drill and carbide dagger drill, as shown in Figure 2. To minimize the effect of tool wear on the machining results, each drill bit was replaced with a new one after processing only four holes. Based on the material characteristics, drill geometry, and previous studies [26,27], a full-factorial experimental design was employed in this study, as summarized in Table 1. This design ensures comprehensive coverage of all parameter combinations and provides a complete dataset for developing reliable quality prediction and multi-objective optimization models.

### 2.2. Determination of Experimental Indicator

#### 2.2.1. Drilling Physical Indicators

**Figure 3:**
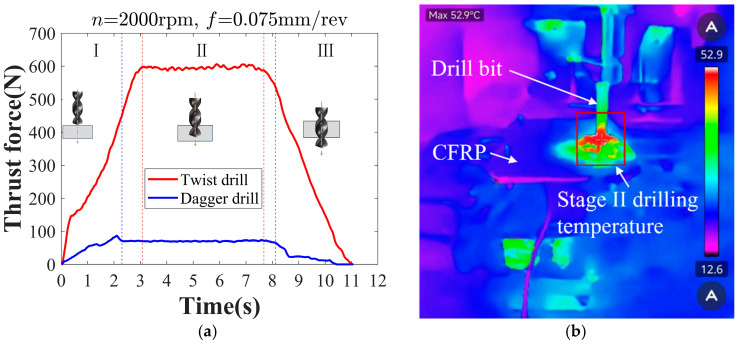
Drilling physical indicators acquisition. (**a**) Thrust force; (**b**) drilling temperature.

The force signal throughout the entire drilling process was measured using the dynamometer shown in Figure 1. In Stage II, the drill has fully penetrated the workpiece as shown in Figure 3a, and the thrust force remains at a relatively stable level with only slight fluctuations. Under these conditions, the measured force can more accurately reflect the impact of tool type and drilling parameters on the thrust force. Therefore, the factor of thrust (*F_T_*) is determined by the average thrust value during the stable drilling stage (Stage II) of the drilling process [28].

Use the thermal infrared camera to collect the temperature data throughout the entire drilling process as shown in Figure 1. When the drilling process enters Stage II, the cutting edges establish stable contact with the material. At this stage, stable friction is maintained between the tool, the chip, and the workpiece, causing the continuous generation of cutting heat, as shown in Figure 3b. Therefore, the temperature measured in Stage II is adopted as the drilling temperature factor (*F_E_*) [29].

#### 2.2.2. Drilling Quality Indicators

**Figure 4:**
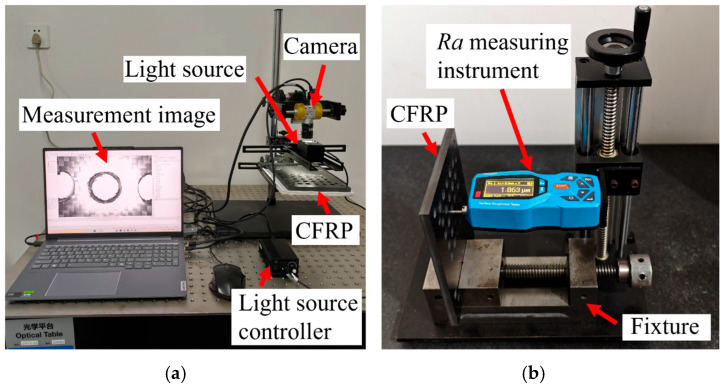
Drilling quality indicators acquisition. (**a**) Delamination and burrs; (**b**) the surface roughness of the hole wall.

The delamination and burrs images of the drilled holes were obtained using the imaging measurement system as shown in Figure 4a.

**Figure 5:**
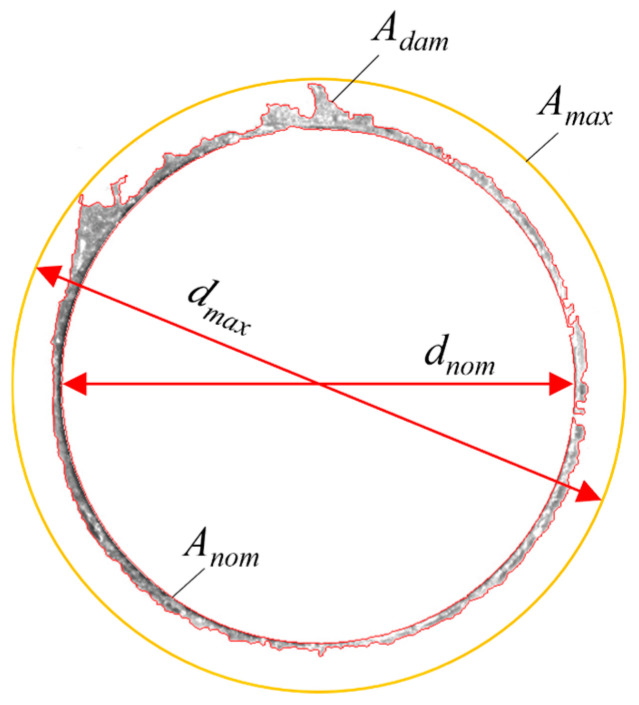
Delamination factor measurement.

The factor of delamination (*F_D_*) is calculated through a weighted combination of the delamination diameter ratio *d_max_*/*d_nom_* and the delamination area ratio *A_max_*/*A_nom_*, where the weighting is determined by the proportion of the delaminated area *A_dam_*/(*A_max_* − *A_nom_*) as shown in Figure 5. This formulation effectively characterizes the extent of drilling delamination damage. The delamination indicator *F_D_* was calculated according to Equation (1) Figure 5 has already been cited in the text [30].

(1) $$F_{D}=(1-\frac{A_{dam}}{A_{max}-A_{nom}})\frac{d_{max}}{d_{nom}}+(\frac{A_{dam}}{A_{max}-A_{nom}})\frac{A_{max}}{A_{nom}}$$

**Figure 6:**
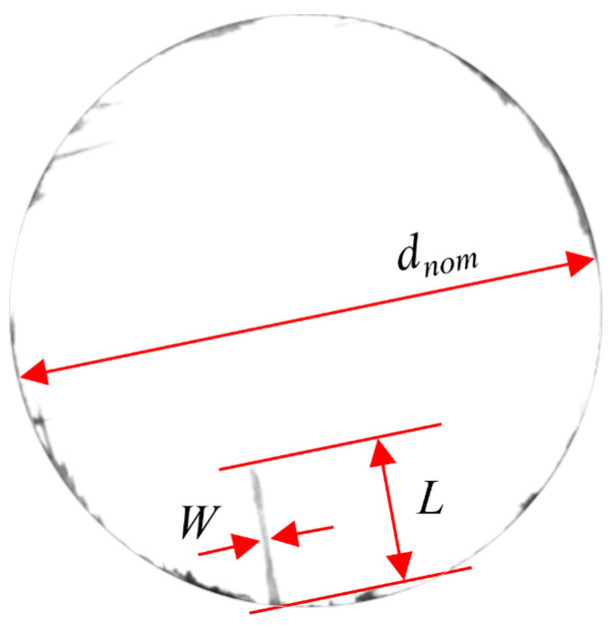
Burr factor measurement.

The burr factor (*F_B_*) is calculated from the maximum radial burr length L, the maximum burr width W, and the nominal hole diameter *d_nom_*, as shown in Equation (2) and Figure 6. In Equation (2), 2*L*/*d_nom_* represents the ratio of the burr length compared to the hole diameter, while *W*/(*πd_nom_*) denotes the coverage ratio of the burr along the hole circumference. Therefore, *F_B_* can effectively characterize the severity of burr formation in drilling [31].

(2) $$F_{B}=\frac{2L}{d_{nom}}+\frac{W}{\pi d_{nom}}$$

The surface roughness of the hole wall is an important indicator for evaluating the drilling quality of CFRP. A surface roughness measuring instrument was used to measure the inner hole wall at multiple locations, and the average value was taken, as shown in Figure 4b.

#### 2.2.3. Material Removal Rate

Material removal rate (MRR) is an essential for evaluating the volume of material removed per unit time during the machining process. MRR is an evaluation parameter of production efficiency in the drilling process. The MRR function is expressed as follows, as shown in Equation (3) [32].

(3) $$MRR=\pi R^{2}nf$$

### 2.3. Analysis of Experimental Results

#### 2.3.1. Analysis of Drilling Physical Indicators

**Figure 7:**
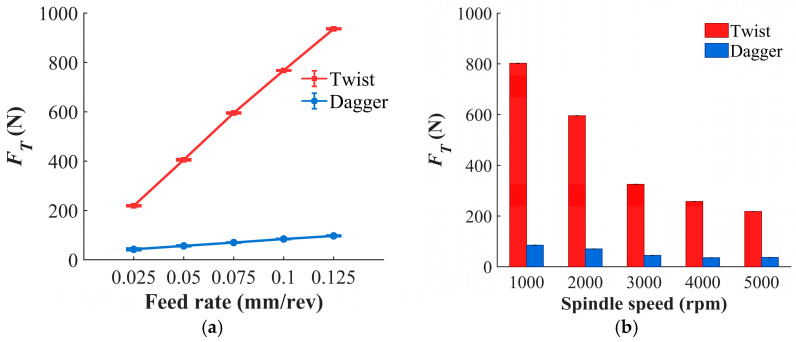
The relationship between *F_T_* and drilling parameters. (**a**) Variation with feed rate at *n* = 2000 rpm; (**b**) variation with spindle speed at f = 0.075 mm/rev.

**Figure 8:**
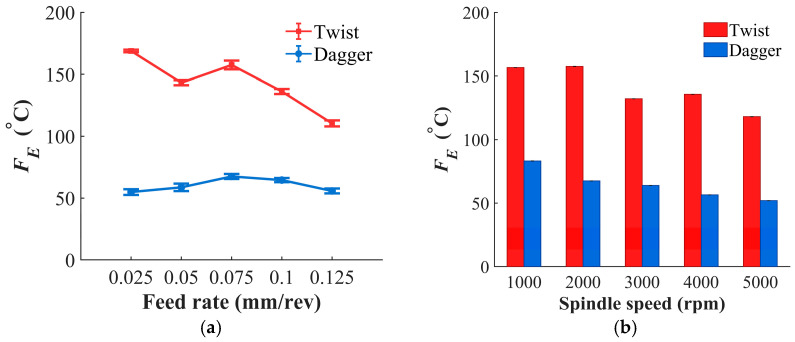
The relationship between *F_E_* and drilling parameters. (**a**) Variation with feed rate at *n* = 2000 rpm; (**b**) variation with spindle speed at *f* = 0.075 mm/rev.

Thrust force and cutting temperature are both physical indicators of the drilling process, as shown in Figure 7. Figure 7a,b shows that, for both the twist drill and the dagger drill, thrust force is positively correlated with feed rate and negatively correlated with spindle speed. Compared with the twist drill, the dagger drill reduces the thrust force by an average of 86%. This is mainly because the dagger drill has a sharper chisel edge and a weaker extrusion effect at the drill center, thereby avoiding the compressive cutting characteristic of the twist drill. As a result, fiber removal is dominated by shear fracture, leading to a much lower axial thrust during drilling. As shown in Figure 8a,b, the cutting temperature of the dagger drill is, on average, 54% lower than that of the twist drill. During drilling, the dagger drill produces a smaller instantaneous contact area involved in friction, which reduces frictional heat generation. In addition, shear-dominated fracture alleviates the secondary friction caused by fiber dragging, further lowering the cutting temperature.

#### 2.3.2. Analysis of Drilling Quality Indicators

**Figure 9:**
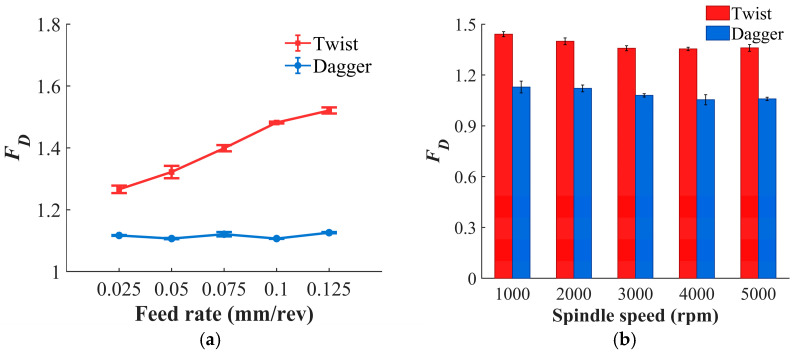
The relationship between *F_D_* and drilling parameters. (**a**) Variation with feed rate at *n* = 2000 rpm; (**b**) variation with spindle speed at *f* = 0.075 mm/rev.

**Figure 10:**
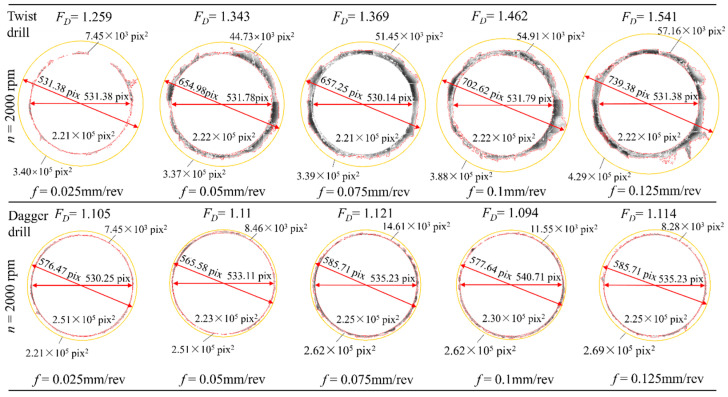
*F_D_* measurement image.

Figure 9 shows the trends of the delamination factor for the two drill types. Measurement image is shown in Figure 10. The delamination factor of the twist drill exhibits a strong positive correlation with feed rate (Figure 9a,b). An increase in feed rate leads to a larger uncut chip thickness per unit time and a higher material removal load, thereby significantly increasing the axial thrust force. The elevated axial thrust makes the interlaminar structure of CFRP more susceptible to damage, resulting in a higher delamination factor. In contrast, the dagger drill has a smaller contact cutting angle with the material, which weakens the extrusion and chisel-edge effects and maintains the axial thrust at a relatively low level. Consequently, its delamination factor is, on average, 20.55% lower than that of the twist drill and is less sensitive to changes in feed rate. In addition, the increase in spindle speed promotes high-speed cutting, which helps alleviate fiber tearing and interlaminar rupture, thereby reducing the occurrence of delamination. Therefore, the delamination factor of both drill types shows a negative correlation with spindle speed.

**Figure 11:**
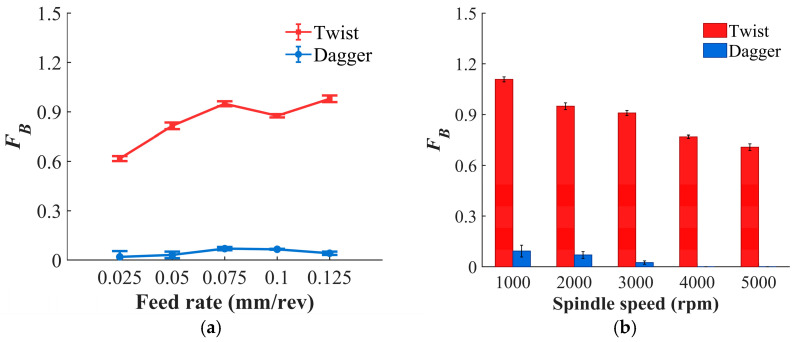
The relationship between *F_B_* and drilling parameters. (**a**) Variation with feed rate at *n* = 2000 rpm; (**b**) variation with spindle speed at *f* = 0.075 mm/rev.

**Figure 12:**
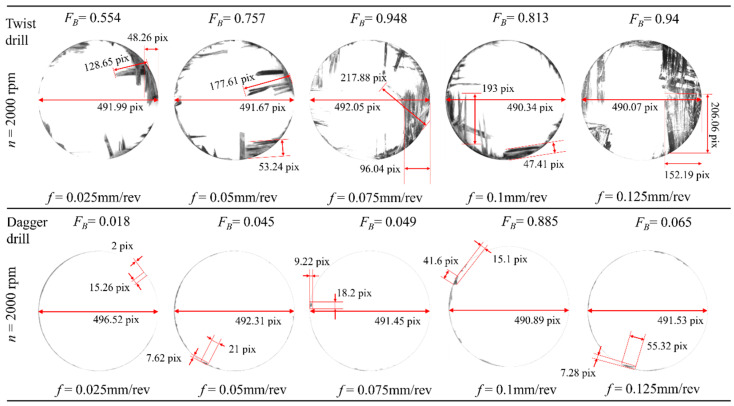
*F_B_* measurement image.

As shown in Figure 11a,b and Figure 12, during drilling with the twist drill, the burr factor increases slightly with feed rate and exhibits noticeable random fluctuations. This is due to the fact that a higher feed rate intensifies the axial extrusion at the hole exit, making the fibers at the exit more prone to bending and tearing, thereby aggravating burrs. In contrast, the dagger drill reduces the burr factor by an average of 95.51%, while only slight fluctuations in burr factor are observed with changes in feed rate. As spindle speed increases, fiber fracture tends to be dominated more by shearing rather than extrusion, which reduces fiber bending and tearing at the hole exit. Therefore, the burr factor for both drill types shows a clear negative correlation with spindle speed. For the twist drill, the shear-dominated cutting effect becomes more pronounced with increasing spindle speed. Owing to its inherent structural advantage in shear cutting, the dagger drill exhibits superior burr suppression performance and can even completely eliminate burrs.

**Figure 13:**
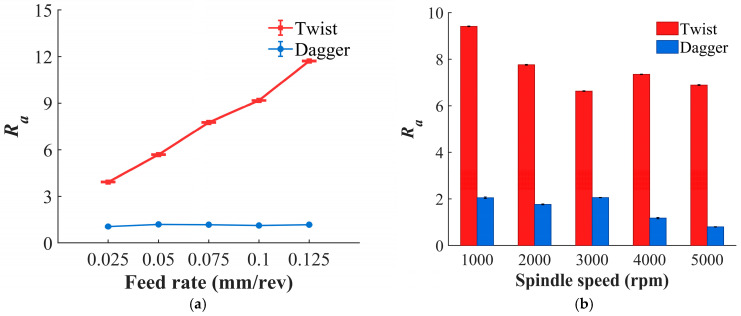
The relationship between *Ra* and drilling parameters. (**a**) Variation with feed rate at *n* = 2000 rpm; (**b**) variation with spindle speed at *f* = 0.075 mm/rev.

The trend of the hole-wall surface roughness is shown in Figure 13. For the twist drill, the hole-wall surface roughness shows a strong positive correlation with feed rate. As the feed rate increases, the indentation effect of the chisel edge on the material becomes more pronounced, making the fibers more likely to bend before tearing. This intensifies fiber pull-out and matrix detachment, thereby resulting in a higher Ra value. In contrast, the dagger drill is relatively insensitive to changes in feed rate. Owing to its shear-dominated cutting mechanism, the hole-wall morphology varies only slightly, and thus no obvious fluctuation in *Ra* is observed. For both drill types, surface roughness generally exhibits a negative correlation with spindle speed. However, for the twist drill, *Ra* increases near 4000 rpm, which is caused by the increase in cutting temperature that causes matrix softening. Once softened, the matrix tends to smear and adhere to the hole wall, thereby deteriorating the surface roughness. By contrast, the dagger drill maintains a consistently lower cutting temperature, making matrix softening less likely to occur. As a result, the hole-wall surface roughness of the dagger drill is, on average, 81.11% lower than that of the twist drill.

Although the above analysis of experimental data can, to some extent, reveal the variation trends of drilling physical and drilling quality indicators with drilling parameters, the drilling process is inherently governed by the coupled effects of multiple parameters, making it difficult to fully reflect the interaction effects among drilling parameters. As a consequence, it is still necessary to establish a regression model under the combined effects of spindle speed and feed rate, which can not only enrich the training data for subsequent predictive modeling, but also provide effective objective and constraint functions for multi-objective optimization.

## 3. Regression Analysis of Drilling Parameters

A polynomial regression equation was used to analyze the relationships between drilling parameters and thrust, drilling temperature, delamination, burr formation, and surface roughness, as shown in Equation (4).

(4) $$Y_{RSM}=a_{0}+\sum_{i=1}^{k}a_{i}x_{i}+\sum_{i=1}^{k}a_{ii}x_{i}^{2}+\sum_{i=1}^{k}\sum_{j=i+1}^{k}a_{ij}x_{i}x_{j}+\varepsilon$$

In the Equation (4), $Y_{RSM}$ represents the response variable for each drilling quality evaluation, $x_{i}$ and $x_{j}$ are the drilling parameters, $a_{0}$, $a_{i}$, $a_{ii}$ and $a_{ij}$ are the coefficients of the polynomial regression function, and ε is the error term.

**Figure 14:**
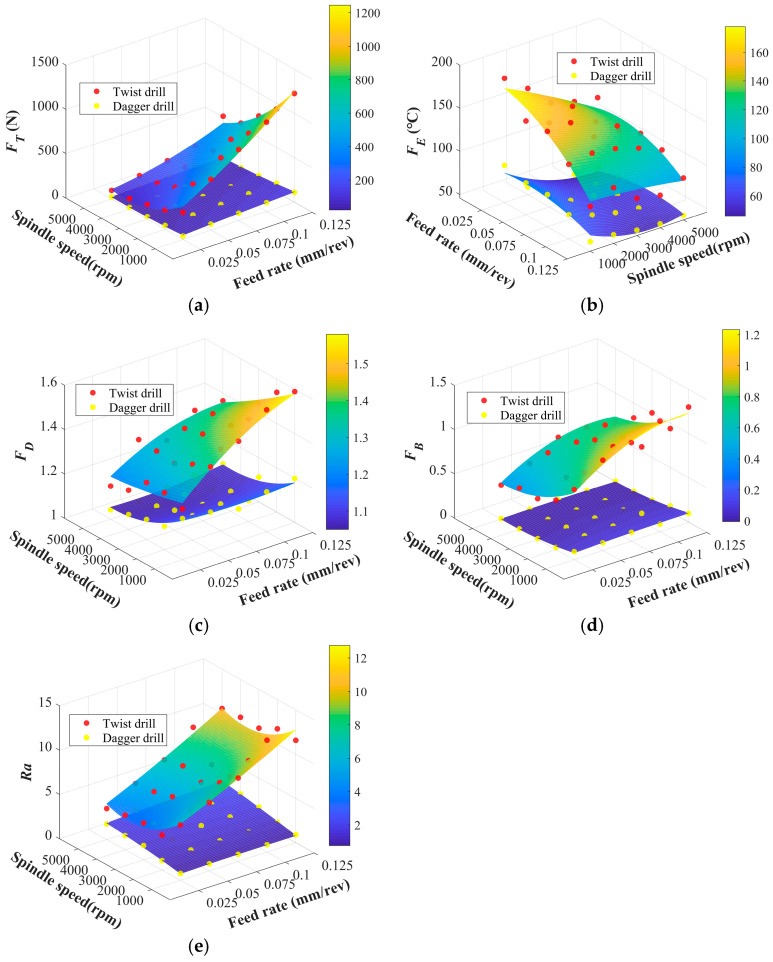
Response surface of drilling parameters. (**a**) Response surface of *F_T_*; (**b**) response surface of *F_E_*; (**c**) response surface of *F_D_*; (**d**) response surface of *F_B_*; (**e**) response surface of *Ra*.

The response surfaces of different drilling parameters are shown in Figure 14.

Among the indicators used for multi-objective optimization, thrust force and the delamination factor were selected as the most critical responses, and their ANOVA results are presented in Table 2 and Table 3.

By combining Figure 14a and Table 2, it can be seen that the twist drill generated substantially higher thrust force than the dagger drill over the tested parameter range. The thrust force increased markedly with increasing feed rate, particularly for the twist drill under low spindle speed and high feed rate conditions. In contrast, the dagger drill maintained a relatively low thrust force and showed lower sensitivity to feed rate variation. This difference can be attributed to the tool-geometry effect. For the twist drill, the chisel edge near the drill center has a very low cutting speed and removes material mainly through extrusion and scraping, resulting in a high axial thrust load. In contrast, the sharper tip and cutting-edge geometry of the dagger drill weaken the extrusion effect and promote a more shearing-dominated material removal mechanism. Consequently, the axial thrust force is reduced, which helps suppress interlaminar separation and improves the drilling quality of CFRP.

Based on Figure 14c and Table 3, the delamination factor of the twist drill is generally higher than that of the dagger drill. For the twist drill, *F_D_* increases significantly with increasing feed rate, particularly under low spindle speed and high-feed-rate conditions. In contrast, the dagger drill maintains a lower *F_D_* over the tested parameter range and shows lower sensitivity to variations in feed rate and spindle speed, indicating its stronger delamination-suppression capability.

This difference is mainly attributed to the tool-geometry effect. Delamination is closely related to the axial thrust generated by feed motion. As the feed rate increases, the twist drill produces a larger axial extrusion load during tool entry and exit, which promotes interlaminar opening, peeling, and crack propagation once the load exceeds the interlaminar bonding strength. By contrast, the dagger drill has a sharper tip and a more favorable cutting-edge structure, which enhances fiber cutting and reduces the axial thrust acting on the uncut laminate. Therefore, the risk of exit delamination and interlaminar crack growth is reduced. This indicates that tool geometry affects not only thrust generation but also the formation mechanism of delamination damage.

## 4. FCE-NN Prediction Model

### 4.1. Principle of the Prediction Model

In order to achieve a comprehensive evaluation of CFRP drilling quality, this study established a comprehensive drilling quality evaluation function based on fuzzification rules [33,34,35]. The function was formed using actual experimental data, response surface analysis results, and expert knowledge from the field regarding the relative importance of different quality defects during drilling as shown in Equation (5). It consists of three key quality indicators: delamination factor, burr factor, and surface roughness.

(5) $$E=W\times S=\left[\begin{array}{ccc} \omega_{1} & \omega_{2} & \omega_{3} \end{array}\right]\times {\left[\begin{array}{ccc} F_{D} & F_{B} & Ra \end{array}\right]}^{T}$$

In Equation (5), *E* denotes the comprehensive evaluation output of drilling quality, *S* represents the evaluation factor of the drilling quality assessment function, and the delamination factor *F_D_*, burr factor *F_B_*, and surface roughness *Ra* are expressed as functions of the feed rate and spindle speed. *W* denotes the weight coefficient matrix of the drilling quality evaluation factors.

Based on expert prior knowledge and the results of response surface analysis, the membership functions of the delamination factor, burr factor, and surface roughness were determined, as shown in Table 4, Table 5 and Table 6.

The weight coefficient matrix *W* of *S* was determined by establishing an expert judgment matrix *A_FCE_* using the 1–9 scale method, which was based on expert knowledge to assess the significance of each quality defect indicator [36].

The eigenvector of the expert judgment matrix *A_FCE_* was calculated and normalized to obtain *W* as shown in Equation (6).

(6) $$W=\left[\begin{array}{ccc} \omega_{1} & \omega_{2} & \omega_{3} \end{array}\right]=\left[\begin{array}{ccc} 0.7396 & 0.1666 & 0.0938 \end{array}\right]$$

**Figure 15:**
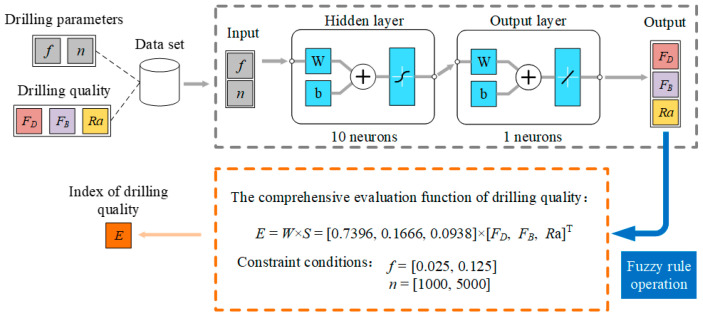
FCE-NN prediction model.

Based on the BP neural network prediction model [37,38], drilling parameters such as feed rate and spindle speed were used as inputs, while drilling quality, including delamination, burr, surface roughness, and the FCE comprehensive evaluation index, was taken as the output (Figure 15).

### 4.2. Prediction Model Performance

**Figure 16:**
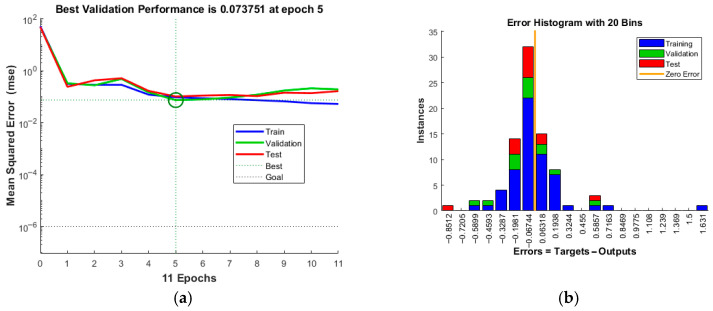
Training process and error analysis of the FCE-NN for delamination: (**a**) Iteration process; (**b**) error histogram.

**Figure 17:**
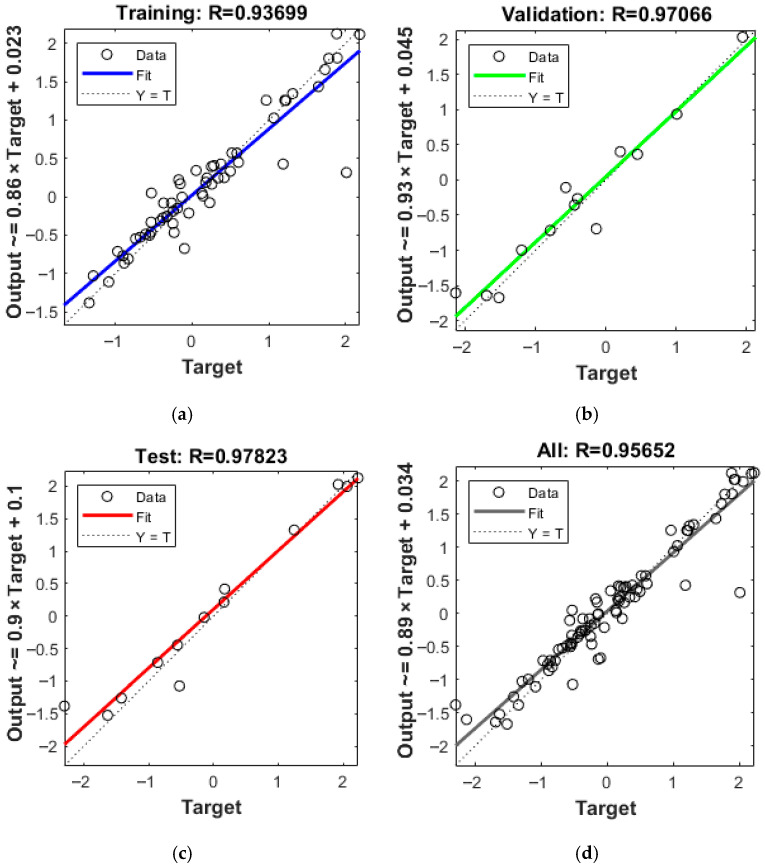
Regression result of FCE-NN for delamination: (**a**) Regression result of the training set; (**b**) regression result of the validation set; (**c**) regression result of the test set; (**d**) regression results for all datasets.

Figure 16 shows the training process and error analysis of the FCE-NN neural network model for delamination prediction. Figure 17 shows the regression results of delamination prediction. The predictive performance of the models for burr formation and surface roughness is shown in Table 7. The model exhibits no overfitting, while the error converges stably, demonstrating high prediction accuracy.

**Figure 18:**
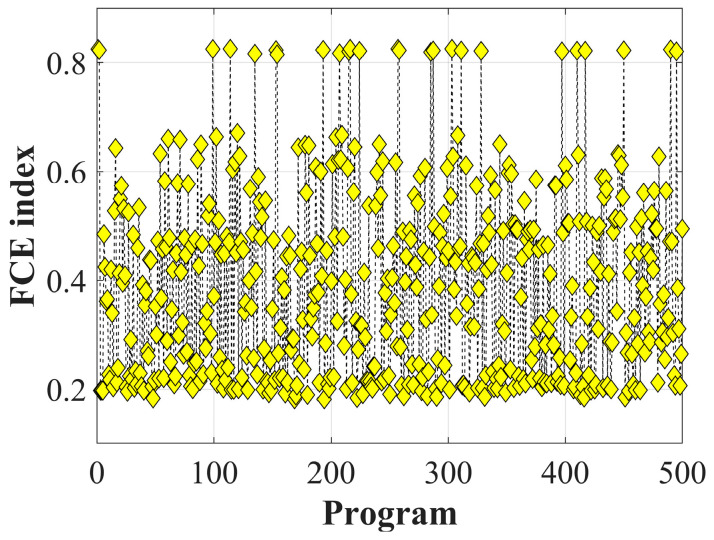
FCE index of drilling quality.

Figure 18 shows the distribution of the FCE index under different drilling schemes. The values are within the interval of 0 to 1, with higher values indicating closer conformity to practical engineering requirements.

## 5. Multi-Objective Optimization of Drilling

In most existing multi-objective optimization studies, drilling-quality indicators are usually treated in parallel, implicitly assuming that they have equal importance. However, this strategy cannot fully reflect the different contributions of various drilling-quality indicators to the overall hole quality.

The multi-objective optimization method proposed in this study combines Pareto-solution generation, objective data-driven ranking, and engineering-oriented comprehensive quality evaluation. First, NSGA-II is used to obtain Pareto-optimal solutions among thrust force, delamination factor, and material removal rate, reflecting the trade-off relationship between drilling quality and machining efficiency. Second, entropy-weighted TOPSIS determines the weights of different indicators according to their data variability and ranks the Pareto solutions objectively. Finally, FCE integrates quality indicators such as *F_D_*, *F_B_*, and *Ra* into a comprehensive drilling-quality index through fuzzy rules, so that the different effects of drilling defects on overall hole quality can be more reasonably considered.

### 5.1. NSGA-II Algorithm

**Figure 19:**
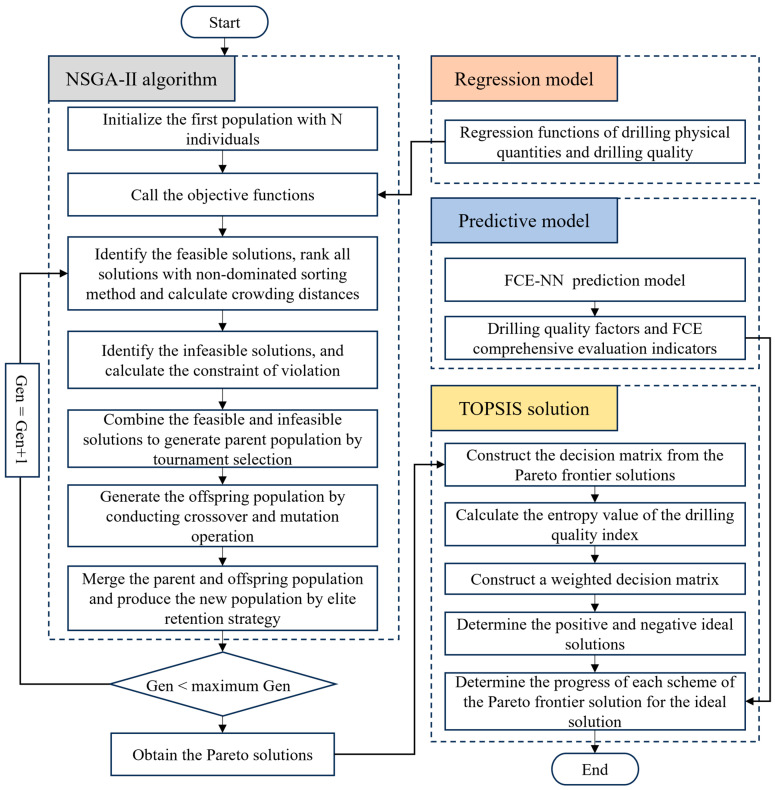
Flowchart of multi-objective optimization.

The NSGA-II algorithm and Pareto front method were used to investigate the multi-objective optimization problem including drilling physical variables, drilling quality, and machining efficiency [39,40]. Specifically, the burr factor and drilling temperature were treated as constraints, while thrust, delamination, and MRR were set as optimization objectives to achieve the ideal solution. The research workflow is illustrated in Figure 19.

The NSGA-II algorithm with additional constraints is adopted as a solution framework for multi-objective optimization involving machining parameters, drilling quality control, and machining efficiency. According to existing studies and multiple experimental trials, the parameters were configured as k = 500, N = 100 N, crossover probability of 0.9, and mutation probability of 0.3.

In Equation (7), the optimization objectives include minimizing thrust, minimizing delamination and maximizing machining efficiency. During the solving process, the algorithm’s constraints should also be designed based on the material’s inherent properties and practical applications, ensuring that drilling quality, drilling temperature, and other relevant factors remain within reasonable ranges as defined in Equation (7).

(7) $$\begin{array}{l} \mathrm{Objective}\;\mathrm{function}:\min(F_{F},\;F_{D},\;-MRR)\\ \mathrm{Constraints}:\left\{\begin{array}{l} 0.025\leq f\leq 0.125\\ 1000\leq n\leq 5000\\ F_{D}\leq 1.4\\ F_{Ra}\leq 11\\ F_{T}\leq T_{g} \end{array}\right. \end{array}$$

### 5.2. FCE Indicator with Entropy Weight TOPSIS Method

A large set of optimal solutions exist in the Pareto front generated by the NSGA-II algorithm, among which drilling schemes suitable for practical engineering applications must be determined. Therefore, a hybrid method is proposed, integrating the entropy weight method, TOPSIS, and FCE. By integrating expert knowledge through FCE with the data-driven results of the entropy weight–TOPSIS method, the method preserves data-driven objectivity while effectively leveraging expert insights to supplement the assessment of complex machining processes, thereby enabling more scientific, robust, and interpretable comprehensive decisions. Calculate the relative closeness of each decision alternative to the ideal solution as shown in Equation (8) [41].

(8) $$O_{i}=\frac{D_{i}^{-}}{D_{i}^{-}+D_{i}^{+}}$$

*O_i_* denotes the relative closeness of the decision option to the ideal solution. A value of *O_i_* approaching 1 indicates a superior performance of the corresponding decision alternative.

In this study, the fuzzy comprehensive evaluation method is incorporated into the relative closeness *O_i_* to the ideal solution, thereby enhancing the assessment of each decision alternative by integrating multiple performance indicators and their fuzzy interactions as shown in Equation (9).

(9) $$O_{FCE}=O_{i}\times E=O_{i}\left[\begin{array}{ccc} \omega_{1} & \omega_{2} & \omega_{3} \end{array}\right]\times {\left[\begin{array}{ccc} F_{D} & F_{B} & Ra \end{array}\right]}^{T}$$

## 6. Results and Discussion

### 6.1. Pareto Optimal Front Results

**Figure 20:**
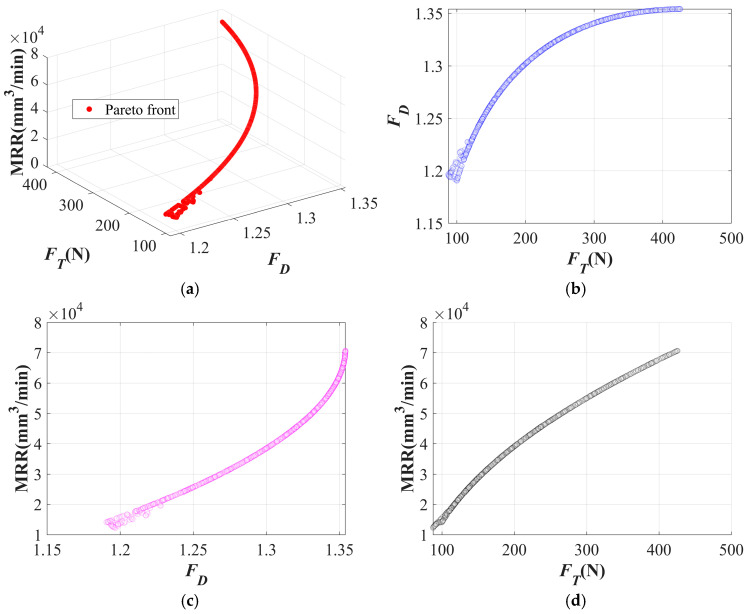
Pareto front of the twist drill: (**a**) Pareto front; (**b**) projection onto the XY plane; (**c**) projection onto the XZ plane; (**d**) projection onto the YZ plane.

The Pareto front of the twist drill is shown in Figure 20a, while Figure 20b–d presents its projected views. In Figure 20b, the *F_D_* shows a clear increasing trend with the increase in *F_T_*. This is because, during drilling with the twist drill, material removal is governed by the combined effects of shearing and extrusion. An increase in thrust force intensifies the extrusion effect, thereby leading to more severe delamination. As shown in Figure 20c,d, pursuing higher machining efficiency tends to result in increased thrust force and delamination.

**Figure 21:**
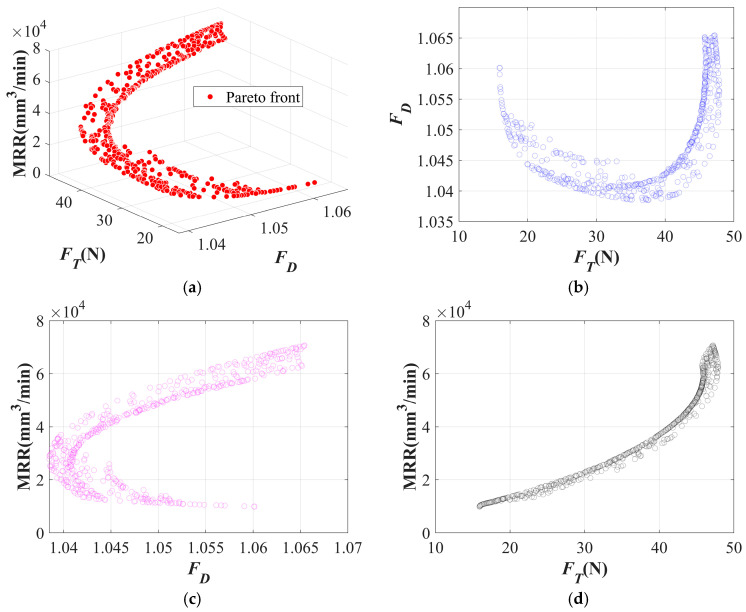
Pareto front of the dagger drill: (**a**) Pareto front; (**b**) projection onto the XY plane; (**c**) projection onto the XZ plane; (**d**) projection onto the YZ plane.

The Pareto front of the dagger drill is shown in Figure 21a, while Figure 21b–d presents its projected views. In Figure 21b, the relationship between *F_T_* and *F_D_* first decreases and then increases. This is because, at relatively low thrust levels, the cutting action of the dagger drill is insufficient, resulting in reduced effective fiber cutting capability and a higher tendency for interlaminar edge tearing. When the thrust force increases to an appropriate range, delamination can be effectively suppressed. However, with a further increase in thrust force, the tip of the dagger drill is still accompanied by an extrusion effect, which aggravates delamination. In Figure 21c,d, the relationship between machining efficiency and delamination is nonlinear, whereas machining efficiency is positively correlated with thrust force.

### 6.2. Multi-Objective Optimization Results

**Figure 22:**
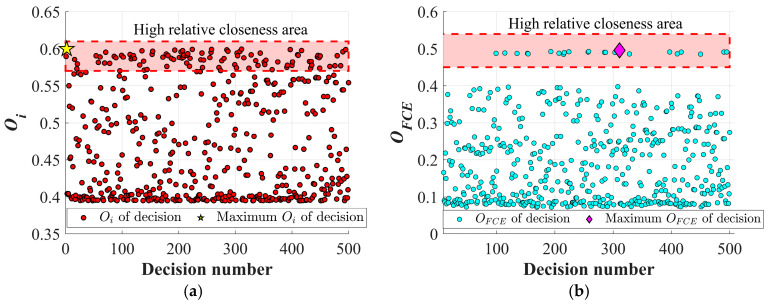
Relative closeness of each decision for the twist drill: (**a**) Relative closeness of each decision without FCE; (**b**) relative closeness of each decision considering FCE.

In Figure 22, the closeness coefficient of each decision scheme obtained with the twist drill is presented. Figure 22a shows the results without considering the FCE index, where the yellow pentagram indicates that the second decision scheme achieves the highest closeness coefficient. In the second decision scheme, *n* is 4999.132 rpm, *f* is 0.0253 mm/rev, *F_T_* is 100.239 N, *F_D_* is 1.191, and the MRR is 14,280 mm^3^/min. Without considering the FCE index, the closeness coefficient of each objective is determined solely by the entropy weight method, and the points in the high correlation progress area are densely distributed. At this stage, the Pareto frontier results do not take into account the extent to which different drilling indicators affect the quality of CFRP drilling holes. Once the FCE index is considered in the evaluation system, the area with high closeness retains the solutions with better overall drilling quality, and the maximum closeness coefficient appears at the 311st decision scheme, as indicated by the pink diamond in Figure 22b. In this case, *n* is 4999.626 rpm, *f* is 0.025 mm/rev, *F_T_* is 99.887 N, *F_D_* is 1.190, and the MRR is 14,140 mm^3^/min. Although the pink diamond shows a decrease in MRR after considering the FCE index, it simultaneously reduces thrust force and further lowers delamination defects, thereby achieving improved hole quality.

**Figure 23:**
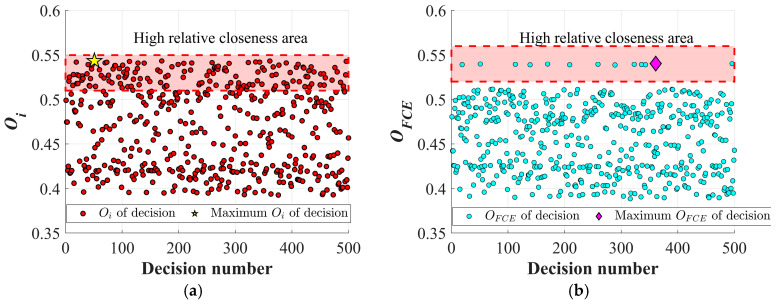
Relative closeness of each decision for the dagger drill: (**a**) Relative closeness of each decision without FCE; (**b**) relative closeness of each decision considering FCE.

**Figure 24:**
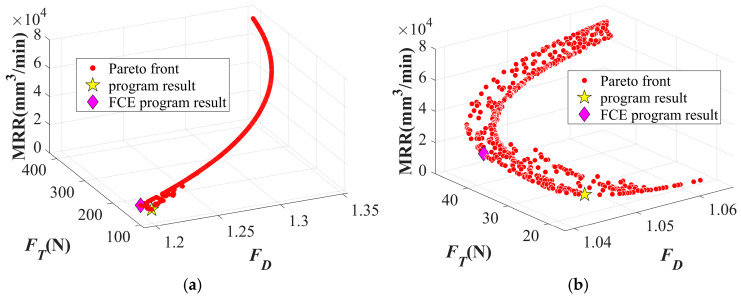
Optimal decision on the Pareto front: (**a**) Pareto optimal decision of twist drill considering FCE; (**b**) Pareto optimal decision of dagger drill considering FCE.

Similarly, the closeness coefficient of each decision scheme obtained with the Dagger drill is shown in Figure 23. Without considering the FCE index, the highest closeness coefficient is observed in the 51st decision scheme, where the *n* is 4422.362 rpm, *f* is 0.0251 mm/rev, *F_T_* is 20.021 N, *F_D_* is 1.044, and the MRR is 12,561.311 mm^3^/min. As shown in Figure 24a, the inclusion of the FCE index leads to a shift in the compromise point of the Pareto front, which aligns more closely with the optimal drilling quality and practical drilling requirements. After considering the FCE index, the maximum closeness coefficient appears at the 361st decision scheme, as indicated by the pink diamond in Figure 23b and Figure 24b. In this case, *n* is 4953.364 rpm, *f* is 0.049 mm/rev, *F_T_* is 36.73 N, *F_D_* is 1.0385, and the MRR is 27,827.795 mm^3^/min.

### 6.3. Experimental Verification Results

Table 8 presents the predicted drilling results of the optimal decision schemes obtained with twist and dagger drills under conditions with and without considering the FCE index. As shown in Table 8, for the twist drill, the introduction of the FCE index reduces *F_T_* from 100.239 N to 99.887 N, while *F_D_*, *F_B_*, and *Ra* decrease by 0.001, 0.002, and 0.017%, respectively. The MRR decreased from 14,280 mm^3^/min to 14,140 mm^3^/min. For the dagger drill, due to its unique tool geometry, after the introduction of the FCE index, f increased from 0.0251 mm/rev to 0.049 mm/rev, and the thrust increased from 20.021 N to 36.73 N as the *f* increased. After the thrust was increased, the *F_D_* decreased by 12.76%. *F_B_* increased slightly by 0.005 and *Ra* increased slightly by 0.255. However, the MRR increased from 12,561 mm^3^/min to 27,827 mm^3^/min. Table 8 further provides an error analysis between the predicted and experimental values.

Analyze the error results. The error of the delamination factor is generally below 5%, indicating that the model can accurately capture the relationship between drilling parameters and delamination damage. The error of surface roughness remains between 1.66% and 6.28%, also demonstrating that the model has good predictive capability for the variation trend of hole-wall quality. The burr factor shows the largest error range, which can be attributed to the complexity and local randomness of burr formation. In particular, when the drill is about to exit the CFRP laminate, the thickness of the uncut layer gradually decreases and the supporting stiffness at the backside of the material becomes weaker. As a result, the fiber bundles are more likely to bend and be pulled out under axial thrust rather than being completely sheared off. This bending and pull-out behavior causes the burr morphology at the hole exit to exhibit irregular and discontinuous characteristics, leading to higher prediction errors than those of the delamination factor and surface roughness.

The prediction errors may be attributed to several factors. In CFRP drilling, the measured responses are affected not only by spindle speed and feed rate, but also by the heterogeneity and anisotropy of the CFRP laminate, local variations in fiber or resin distribution, slight tool-to-tool differences, tool wear, chip-removal instability, transient thermo-mechanical coupling, and the initiation and propagation of drilling-induced damage. These factors make the real drilling process more complex than the relationships captured by the prediction model. In addition, errors may also arise during thrust-force acquisition, infrared temperature measurement, defect image processing, delamination and burr boundary extraction, and surface roughness measurement. Nevertheless, the overall errors remain within an acceptable range, indicating that the proposed model can effectively capture the dominant variation trends of drilling-quality indicators and demonstrates good predictive capability and engineering applicability.

## 7. Conclusions

This study investigated the drilling quality of CFRP through analysis, prediction, and optimization using two different drill geometries. The main conclusions are as follows:

- Drilling experiments on CFRP were conducted using a twist drill and a dagger drill. The characteristics and differences in drilling physical indicators and drilling quality indicators under individual drilling parameters were analyzed. Compared with the twist drill, the dagger drill reduced the delamination factor, burr factor, and surface roughness by 20.6%, 95.5%, and 81.1% on average, respectively.
- For both the twist drill and the dagger drill, regression models were established for thrust force, drilling temperature, delamination, burr formation, and hole-wall surface roughness under multiple drilling parameters, and the variation patterns under the combined effects of spindle speed and feed rate were analyzed. The coefficients of determination of the regression models were 0.98 and 0.94 for *F_T_*, 0.87 and 0.84 for *F_E_*, 0.94 and 0.92 for *F_D_*, 0.90 and 0.92 for *F_B_*, and 0.90 and 0.94 for *Ra*, indicating that the established models can effectively explain the variation trends of the drilling response indicators and possess high fitting accuracy and reliability.
- An FCE-NN prediction model was proposed, in which different drilling quality indicators were quantified through fuzzy rules to reflect the severity of drilling-induced defects. The prediction accuracies for *F_D_*, *F_B_*, and *Ra* reached 95.61%, 95.46%, and 83.36%, respectively, while also providing a comprehensive quality evaluation index.
- The NSGA-II algorithm was employed for multi-objective optimization, and the Pareto-front solutions were further ranked using the FCE index combined with entropy-weighted TOPSIS. The optimized drilling parameters were n = 4999.626 rpm and f = 0.025 mm/rev for the twist drill, and n = 4953.364 rpm and f = 0.049 mm/rev for the dagger drill. Actual drilling validation experiments were conducted under these optimized conditions. For the twist drill, the predicted values of *F_D_*, *F_B_*, and *Ra* were 1.190, 0.412, and 3.946, respectively, while the corresponding actual experimental real detection values were 1.134, 0.451, and 4.177. The prediction errors were 4.90%, 8.65%, and 5.53%, respectively. For the dagger drill, the predicted values of *F_D_*, *F_B_*, and Ra were 1.039, 0.009, and 2.057, respectively, while the corresponding actual experimental real detection values were 1.062, 0.0087, and 2.001. The prediction errors were 2.21%, 3.33%, and 2.89%, respectively.

## Figures and Tables

**Table 1:** Experimental data of twist drills and dagger drills.

Tool	*f* (mm/rev)	*n* (rpm)	*F_T_* (N)	*F_E_* (°C)	*F_D_*	*F_B_*	*Ra*	*MRR*
Twist drill	0.025	1000	306.22	189.7	1.229	0.790	6.25	2827.43
	0.025	2000	219.21	168.8	1.256	0.617	3.92	5654.87
	0.025	3000	167.23	143.2	1.257	0.455	4.16	8482.3
	0.025	4000	119.87	135.6	1.176	0.461	3.9	11,309.73
	0.025	5000	94.96	131.2	1.149	0.381	3.51	14,137.17
	0.05	1000	573.31	154.4	1.377	1.016	7.72	5654.87
	0.05	2000	405.66	143.2	1.343	0.815	5.69	11,309.73
	0.05	3000	253.84	153.3	1.3	0.592	6.08	16,964.6
	0.05	4000	185.53	126.3	1.311	0.756	5.49	22,619.47
	0.05	5000	156.29	107.2	1.316	0.519	5.26	28,274.33
	0.075	1000	802.09	156.6	1.45	1.108	9.41	8482.3
	0.075	2000	595.16	157.5	1.369	0.949	7.76	16,964.6
	0.075	3000	325.5	132.2	1.391	0.909	6.63	25,446.9
	0.075	4000	257.68	135.7	1.37	0.769	7.35	33,929.2
	0.075	5000	218.13	118.1	1.27	0.708	6.89	42,411.5
	0.1	1000	1007.06	132.3	1.55	1.246	12.64	11,309.73
	0.1	2000	767.55	136.2	1.462	0.876	9.18	22,619.47
	0.1	3000	585.5	132.4	1.388	0.824	6.23	33,929.2
	0.1	4000	340.36	124	1.395	0.856	6.41	45,238.93
	0.1	5000	282.27	112.8	1.364	0.718	9.5	56,548.67
	0.125	1000	1224.11	97.8	1.59	1.430	11.57	14,137.17
	0.125	2000	936.2	110.4	1.541	0.979	11.71	28,274.33
	0.125	3000	742.08	89.1	1.446	1.005	10.64	42,411.5
	0.125	4000	546.73	83.5	1.396	0.833	10.72	56,548.67
	0.125	5000	615.24	94.5	1.365	0.667	10.56	70,685.83
Dagger drill	0.025	1000	37.91	88.8	1.186	0.088	1.772	2827.43
	0.025	2000	42.92	54.9	1.117	0.02	1.576	5654.87
	0.025	3000	28.87	50.3	1.09	0.004	3.02	8482.3
	0.025	4000	19.52	51.2	1.097	0	1.057	11,309.73
	0.025	5000	23.38	50.3	1.042	0	0.765	14,137.17
	0.05	1000	66.85	82.5	1.172	0.08	2.619	5654.87
	0.05	2000	56.78	58.7	1.107	0.032	1.925	11,309.73
	0.05	3000	37.37	52.5	1.089	0.027	1.217	16,964.6
	0.05	4000	28.31	54.1	1.062	0	1.198	22,619.47
	0.05	5000	29.49	53.2	1.036	0	0.79	28,274.33
	0.075	1000	85.16	83.2	1.129	0.093	2.05	8482.3
	0.075	2000	70.33	67.5	1.121	0.070	1.764	16,964.6
	0.075	3000	44.74	63.9	1.08	0.025	2.058	25,446.9
	0.075	4000	35.86	56.5	1.054	0	1.176	33,929.2
	0.075	5000	36.97	52	1.059	0	0.8	42,411.5
	0.1	1000	100.11	74.1	1.179	0.08	3.938	11,309.73
	0.1	2000	84.68	64.6	1.107	0.066	1.971	22,619.47
	0.1	3000	48.16	58.3	1.09	0.009	2.493	33,929.2
	0.1	4000	38.57	54.7	1.049	0	1.125	45,238.93
	0.1	5000	43.5	53.3	1.073	0	0.83	56,548.67
	0.125	1000	109.46	56.8	1.198	0.095	2.204	14,137.17
	0.125	2000	97.33	55.8	1.126	0.042	1.566	28,274.33
	0.125	3000	69.55	52.1	1.112	0.016	2.148	42,411.5
	0.125	4000	43.39	50.3	1.06	0	1.176	56,548.67
	0.125	5000	47.97	51.2	1.08	0	0.904	70,685.83

**Table 2:** ANOVA results for *F_T_*.

Source	Twist Drill			Dagger Drill		
	Sum of Squares	F-Value	*p*-Value	Sum of Squares	F-Value	*p*-Value
*f*	1,128,557.81	701.3	1.84 × 10^−16^	5542.36	128.93	0.02
*n*	918,123.61	570.53	1.23 × 10^−15^	7756.35	180.44	0.002
*f n*	116,506.17	72.4	6.63 × 10^−8^	930.62	21.65	0.109
*f* ^2^	8673.83	5.39	0.03	22.11	0.51	0.261
*n* ^2^	47,690.44	29.64	2.98 × 10^−5^	495.05	11.56	0.015
Residual	30,575.54			816.75		
Total	2,250,127.4			15,565.23		
R^2^	0.98			0.94		

**Table 3:** ANOVA results for *F_D_*.

Source	Twist Drill			Dagger Drill		
	Sum of Squares	F-Value	*p*-Value	Sum of Squares	F-Value	*p*-Value
*f*	0.19	67.1	1.84 × 10^−11^	0.0006	2.51	0.13
*n*	0.064	38.701	1.18× 10^−7^	0.04	179.74	3.9 × 10^−11^
*f n*	0.001	10.4	0.0045	0.0002	0.85	0.37
*f* ^2^	0.007	7.27	0.014	0.0006	2.76	0.11
*n* ^2^	0.0003	0.28	0.6	0.0048	22	0.0002
Residual	0.018			0.0042		
Total	0.29			0.05		
R^2^	0.94			0.92		

**Table 4:** The membership function of the *F_D_*.

Level	Delamination Factor	Scale	Membership Function
Low	*F_D_* < 1.2	0.9	1
Medium	1.2 ≤ *F_D_* < 1.4	0.7	(1.4 − *x*)/0.2
	1.4 ≤ *F_D_* < 1.6	0.5	(1.6 − *x*)/0.2
High	1.4 ≤ *F_D_* < 1.8	0.3	(1.8 − *x*)/0.2
	*F_D_* > 1.8	0	0

**Table 5:** The membership function of the *F_B_*.

Level	Delamination Factor	Scale	Membership Function
Low	*F_B_* < 0.3	0.9	1
Medium	0.3 ≤ *F_B_* < 0.6	0.7	(0.6 − *x*)/0.3
	0.6 ≤ *F_B_* < 0.9	0.5	(0.9 − *x*)/0.3
High	0.9 ≤ *F_B_* < 1.2	0.3	(1.2 − *x*)/0.3
	*F_B_* > 1.2	0	0

**Table 6:** The membership function of the *R_a_*.

Level	Delamination Factor	Scale	Membership Function
Low	*Ra* < 3	0.9	1
Medium	3 ≤ *Ra* < 6	0.7	(0.6 − *x*)/0.3
	6 ≤ *Ra* < 9	0.5	(0.9 − *x*)/0.3
High	9 ≤ *Ra* < 12	0.3	(1.2 − *x*)/0.3
	*Ra* < 12	0	0

**Table 7:** Model prediction performance.

Quality	Best Epoch	Training R	Validation R	Test R	All R
F_D_	11	0.94	0.97	0.98	0.96
F_B_	25	0.95	0.98	0.97	0.95
Ra	11	0.84	0.78	0.89	0.83

**Table 8:** Predicted and experimental values of drilling quality.

	Twist	Dagger	Twist with FCE Index	Dagger with FCE Index
*n* (rpm)	4999.132	4422.362	4999.626	4953.364
*f* (mm/rev)	0.0253	0.0251	0.0250	0.049
MRR (mm^3^/min)	14,280	12,561	14,140	27,827
*F_T_* (*N*)	100.239	20.021	99.887	36.73
*F_D_* (Pred)	1.191	1.044	1.190	1.039
*F_D_* (Act)	1.14	1.073	1.134	1.062
*F_D_* Error (%)	4.47%	2.69%	4.94%	2.21%
*F_B_* (Pred)	0.414	0.004	0.412	0.009
*F_B_* (Act)	0.469	0.0043	0.451	0.0087
*F_B_* Error (%)	11.73%	7.5%	8.65%	3.33%
*R_a_* (Pred)	3.963	1.802	3.946	2.057
*R_a_* (Act)	4.162	1.772	4.177	2.001
*R_a_* Error (%)	6.28%	1.66%	5.53%	2.8%

## Data Availability

The original contributions presented in this study are included in the article. Further inquiries can be directed to the corresponding author.
